# Somatic Mutations and Clonal Hematopoiesis as Drivers of Age-Related Cardiovascular Risk

**DOI:** 10.1007/s11886-022-01724-2

**Published:** 2022-06-03

**Authors:** Bernhard Haring, Stephanie Wissel, JoAnn E. Manson

**Affiliations:** 1 Department of Medicine III, Saarland University Hospital, Homburg, Saarland Germany; 2 Department of Medicine I, University of Würzburg, Würzburg, Bavaria, Germany; 3 Department of Epidemiology and Population Health, Albert Einstein College of Medicine, Bronx, NY USA; 4 Department of Medicine, Brigham and Women’s Hospital, Harvard Medical School, Boston, MA USA; 5 Department of Epidemiology, Harvard T.H. Chan School of Public Health, Boston, MA USA

**Keywords:** Somatic mutations, Clonal hematopoiesis of indeterminate potential, Age-related cardiovascular risk, Preventive cardiology

## Abstract

**Purpose of Review:**

Clonal hematopoiesis of indeterminate potential (CHIP) has been identified as a novel cardiovascular risk factor. Here we review the relationship of lifestyle and environmental risk factors predisposing to somatic mutations and CHIP and provide an overview on age-related cardiovascular outcomes.

**Recent Findings:**

CHIP has been associated with accelerated atherosclerosis and cardiovascular disease in both epidemiological and experimental studies. The most commonly mutated candidate driver genes are *DNMT3A*, *TET2*, *JAK2*, and *ASXL1*. The underlying mechanisms appear predominantly related to inflammatory pathways. Although age is the dominant risk factor for developing CHIP, emerging evidence suggests that other factors such as smoking, obesity/type 2 diabetes, or an unhealthy diet play a role in the occurrence of somatic mutations.

**Summary:**

Evidence suggests a strong link between vascular risk factors, somatic hematopoietic mutations, and age-related cardiovascular disease. Further studies on CHIP biology are required to identify targeted interventions for risk reduction in patients with CHIP and inform the utility of screening strategies.

## Introduction

The human immune system relies upon hematopoietic stem cells (HSCs), which are precursors to erythroid, lymphoid, and myeloid cells and platelets that regulate immunity and inflammation.

Due to a combination of genetic predisposition, environmental exposures, and random chance, some HSCs acquire specific somatic mutations with leukemogenic potential, which result in cellular survival advantages and clonal expansion of cells in that lineage. This phenomenon, the clonal expansion of HSCs harboring leukemogenic mutations in the absence of other criteria for hematologic neoplasia, dysplasia, or cytopenia, is termed clonal hematopoiesis of indeterminate potential (CHIP) [1, 2].

Human aging is associated with an increased frequency of somatic mutations in HSCs over the lifetime. The prevalence of CHIP in peripheral blood is low (< 0.5%) from birth until 50 years of age after which it begins to rise, affecting 10% of persons aged 70 to 80 years [3]. Most patients with CHIP have somatic mutations in regulator or DNA repair genes such as *DNMT3A*, *TET2*, or *ASXL1*, which increase in frequency with age [4••, 5]. Although such somatic mutations greatly increase the risk of acquiring additional driver mutations resulting in a 10- to 100-fold increased relative risk of hematologic malignancy, the main cause of death in individuals with CHIP is atherosclerotic cardiovascular disease (CVD). The absolute risk for acquiring a hematologic malignancy remains modest (0.5 to 1% per year) [6].

In this review, we first address the relationship of selected cardiovascular risk factors with CHIP and describe the most commonly found somatic mutations. Second, we provide an overview on age-related cardiovascular outcomes related to the presence of CHIP. Finally, we provide our perspective on the potential clinical utility of screening for CHIP for CVD prevention. 

## Cardiovascular Risk Factors and CHIP

**Fig. 1:**
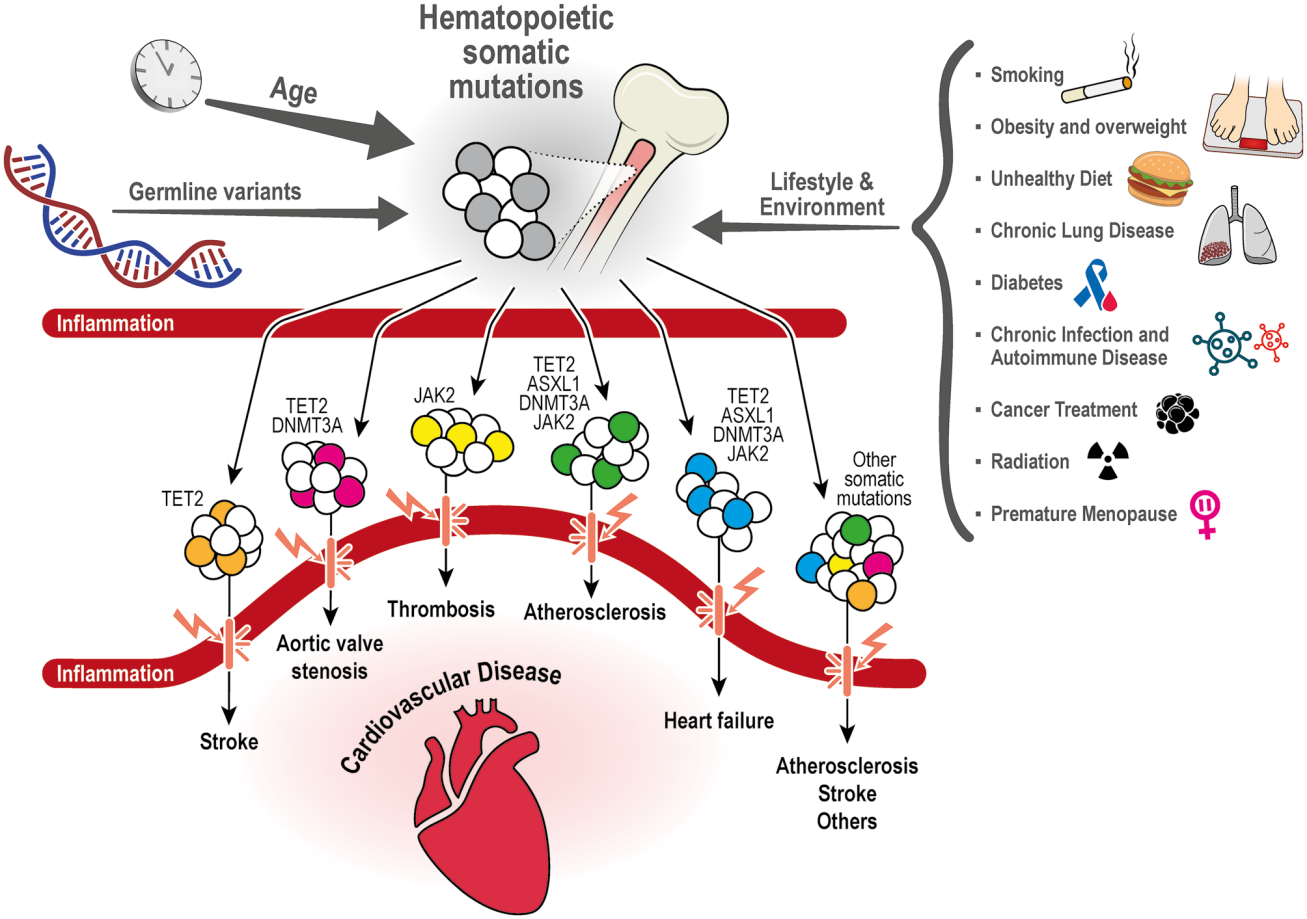
Induction of somatic mutations, clonal hematopoiesis, and cardiovascular risk

Age is the dominant risk factor for CHIP, which parallels other chronic diseases of aging [6]. Emerging evidence suggests that certain environmental factors and lifestyle exposures may play a role in the induction of somatic mutations and the development of CHIP [6–8] (Fig. 1). Adherence to a healthy lifestyle is a major approach to controlling CVD, associated with more favorable CVD risk factor profiles and with lower CVD incidence and mortality [9–12]. Because many CVD risk factors are influenced by lifestyle, modifiable behavioral factors may also be associated with a lower presence of CHIP. Although research is still limited on which CVD risk factors are related to CHIP, we briefly summarize the available evidence below.

### Smoking and Chronic Obstructive Lung Disease

Smoking has been positively associated with presence of CHIP, although findings have not been uniform. [5, 13–16]. Prior inconsistencies are most likely explained by active as compared to former smoking status. Using data from the UK Biobank, a smoking history was significantly associated with CHIP [17]. However, this association was largely driven by those who were current smokers rather than former smokers. Interestingly, among specific CHIP mutations, *ASXL1* mutations seem to be particularly enriched with current and past smokers [17]. In line with these findings, individuals with CHIP were recently shown to be at significantly higher risk of compared to non-carriers [18]. Moreover, smoking exposure was found to be associated with a small but significantly increased risk of having CHIP. Detailed analysis further showed that inactivation of TET2 was associated with the development of emphysema and inflammation in models using cigarette smoke exposure [18]. Most recent evidence stems from two-sample Mendelian randomization analyses [16], showing that smoking is strongly associated with mosaic chromosomal alterations but not with CHIP. These recent findings support a causal association between smoking and mosaic chromosomal alterations and suggest that smoking may variably shape the fitness of clones bearing somatic mutations [16].

### Diet

The relationship between diet quality and presence of CHIP has yielded conflicting results [19, 20]. While some authors did not find any association, other results suggest that an unhealthy diet may be associated with a higher prevalence of CHIP [19, 20]. In an analysis using data from a large cohort of postmenopausal women, Haring et al. did not find a relationship between adherence to a healthy diet (assessed by the Alternative Healthy Eating Index-2010) and prevention of major chronic diseases and presence of CHIP [19]. On the other hand, recent evidence stemming from the UK Biobank suggests that an unhealthy diet quality as defined by intake of fruits and vegetables, red meat, processed food, and added salt is associated with a higher prevalence of CHIP and higher rates of adverse CVD events and death independent of CHIP status [20]. Differences may be explained by study population characteristics, measurement errors, or other factors and warrant further clarification.

### Obesity and Type 2 Diabetes

Adipose tissue can synthesize cytokines such as TNF-α and IL-6 and has been shown to promote inflammation and atherogenesis independent of effects on insulin resistance or lipoproteins [21]. Having a normal body mass index compared to being obese is associated with lower frequency of CHIP in postmenopausal women [19]. Jaiswal et al. reported a 1.3-fold increased odds of CHIP in diabetes patients [22]. Obesity, diabetes, and CHIP may be related to one another through increased activation of pro-inflammatory pathways [21]. In fact, *DNMT3A* and *TET2* may mediate atherosclerotic cardiovascular risk through regulating lipid and glucose metabolism [15, 23].

*DNMT3A* is significantly increased in adipose tissue–derived macrophages in mice fed with high-fat diet [24]. Similarly, a recent report by Fuster et al. indicates that clonal expansion in *TET2* deficient cells can aggravate insulin resistance, obesity, and aging in mice [25]. Thus, a combination of aging, adipose tissue accumulation, and CHIP-associated mutations might activate the production of inflammatory cytokines. In agreement with this hypothesis, *TET2*-deficient HSCs have been shown to produce increased levels of monocytes and inflammatory cytokines such as IL-1β and IL-6 [26, 27]. In fact, serum levels of IL-6 are generally high in people with CHIP, paralleling the observation that obesity or aging also induce an inflammatory response in the bone marrow by promoting accumulation of adipocytes [4••, 28, 29].

A positive feedback loop between self-renewal of HSCs and progression of inflammation in *TET2*-mutated CHIP has been proposed [25, 27]. *TET2*-mutated HSCs preferentially produce myeloid progenitors. As a result, monocytes and macrophages are increased. In macrophages with *TET2* mutations, the NLRP3 inflammasome pathway is activated and IL-1β is increasingly secreted. This eventually leads to an overproduction of IL-1β in the adipose tissue explaining a relationship between CHIP and type 2 diabetes mellitus as the IL-1β-mediated autoinflammatory process is regarded as a major factor in the loss of beta-cell mass in type 2 diabetes [25, 30]. In turn, IL-1β promotes the self-renewal of HSCs with *TET2* mutations. This positive feedback loop would be responsible for the pathogenesis of diabetes and atherosclerosis in *TET2*-mutated CHIP [27].

### Hyperlipidemia

Chronic elevation of blood lipid levels in conjunction with immune cell recruitment and inflammation accelerates the development of atherosclerotic plaques. Interestingly, however, CHIP mutations have not been not associated with lipid levels apart from the association with *JAK2* CHIP, which is correlated with a decrease in total cholesterol and LDL despite an increased risk for coronary heart disease [4••, 8]. The lack of a clear relationship between most CHIP mutations and hyperlipidemia has been a surprising finding. Insights were recently provided by Heyde et al. who demonstrated that mild hypercholesterolemia in non-atherosclerotic wild-type mice did not induce clonal expansion, suggesting that in the absence of inflammation, elevated cholesterol alone is not sufficient to drive clonal hematopoiesis [31••].

### Premature Menopause

A history of premature menopause was found to be independently associated with increased odds of CHIP in two large cohorts of postmenopausal women [32]. Moreover, in gene-specific analyses, only *DNMT3A* was significantly associated with premature menopause. Interestingly, the risks of developing CHIP appeared to differ in women with natural versus surgical premature menopause, implying that postmenopausal reductions in estrogen and other sex steroid hormones alone may not explain the relationship between premature menopause and CHIP. Independent of established CHIP risk factors, premature menopause was associated with 1.4-fold odds—and natural premature menopause with 1.7-fold odds—of CHIP [32].

### HIV and Chronic Infection

HIV infection is associated with greater risk for hematologic malignancy and coronary artery disease is a major cause of morbidity. Bick et al. showed that in individuals living with HIV, the prevalence of CHIP is increased twofold compared to matched controls. Interestingly, ASXL1 was the most commonly implicated mutated CHIP gene [33].

### Cancer Treatment and Radiation

Bolton et al. examined the effects of different cancer therapies on CHIP [34]. Those most associated with CHIP prevalence were external beam radiation therapy, cytotoxic chemotherapy, and radionuclide therapy. Within cytotoxic chemotherapy, topoisomerase II inhibitors (e.g., doxorubicin) had the strongest association along with platinum agent carboplatin. CHIP has also been associated with environmental radiation. Recently presented analyses from WHI suggest ambient exposure to radon was associated with CHIP prevalence [35]. Furthering the work on ambient radiation and CHIP, Mencia-Trinchant et al. conducted a unique study on a pair of twin astronauts using data from the NASA Twins Study [36•]. These astronauts exhibited CHIP almost two decades prior to the mean age at which it is typically detected and showed larger shifts in clone size than age-matched controls or radiotherapy patients.

## Somatic Mutations and CHIP

The most commonly mutated candidate driver genes in CHIP are *DNMT3A*, *TET2*, and *ASXL1* (Table 1) [8, 15]. These three somatic mutations account for 75% of all CHIP cases [4••]. Additional mutations are seen in *JAK2*, which is particularly associated with increased rates of thrombosis, as well as the DNA damage response pathway genes *PPM1D* and *TP53*, and mRNA splicing factors *SRSF2* and *SF3B1*. Early analysis found mutations in *DNMT3A*, *TET2*, and *ASXL1* to be associated with a 1.7-fold to 2.0-fold increased risk of incident coronary heart disease, while the *JAK2 V617F* mutation was associated with a 12-fold increased risk [15]. The vast majority of individuals (approximately 90%) with CHIP driver mutations have only one identified mutation. Across age groups, *JAK2* CHIP carriers are the youngest. Relative to *JAK2*, *ASXL1*, and *TET2* carriers are 3.3 and 3.9 years older, while *PPM1D*, *SF3B1*, and *SRSF2* carriers are 5.0, 6.9, and 7.7 years older, respectively [4••].

**Table 1:** Common CHIP driver mutations by cardiovascular risk factor or outcome event

	Common CHIP driver mutations				
	TET2	DNMT3A	ASXL1	JAK2	Reference
*Cardiovascular risk factor*					
Smoking			x		[5, 16–19, 53]
Obesity and/or diabetes	x	x			[14, 19, 23]
Unhealthy diet	x	x			[19, 20]
Hypercholesterolemia	x			x	[4••, 31••, 76]
Premature menopause		x			[32]
HIV infection			x		[33, 77]
*Cardiovascular outcome event*					
Coronary heart disease	x	x	x	x	[15]
Heart failure	x	x	x	x	[13, 63, 78]
Stroke	x				[58]
Aortic valve stenosis	x	x			[57]

### DNMT3A

With a frequency of approximately 50% in all CHIP cases, DNA methyltransferase 3a (*DNMT3A*) is considered to be the most commonly mutated gene in CHIP [4••]. *DNMT3A* represents an epigenetic regulator of gene expression and encodes a methyltransferase enzyme that catalyzes DNA methylation. Pathogenic mutations of *DNMT3A* promote HSC self-renewal and the expression of multipotency genes while suppressing differentiation factor expression. This enables *DNMT3A* mutations to affect all hematopoietic lineages, inducing pro-inflammatory T-cell polarization and activating the inflammasome complex.

Using CRISPR gene technology, it was possible to show in mouse models that *DNMT3A* CHIP can both cause inflammation and be promoted by inflammation itself [37]. Murine macrophages carrying *DNMT3A* mutations showed increased expression of several cytokines. On the other hand, interferon-gamma is sufficient to drive clonal expansion of *DNMT3A* mutant HSCs, in which via increased resistance to stress-induced apoptosis and differentiation, defects can easily outcompete wild-type HSCs in peripheral blood.

### TET 2

With a presence of approximately 20% in all CHIP cases, *TET2* is the second most frequently mutated gene in CHIP. *TET2* acts in antagonistic fashion to *DNMT3A* by catalyzing the oxidation of the DNA-methyl group (demethylation) and also affects transcription by recruiting histone modifiers. *TET2* loss-of-function mutations cause epigenetic dysregulation, promote HSC self-renewal, and preferentially lead towards myeloid lineage differentiation [38].

Analyses of macrophages from mice that received bone marrow with *TET2* deficient cells showed elevated expression of several chemokine and cytokine genes that contribute to a pro-inflammatory state and accelerated atherosclerosis [15]. *TET2* deficient carriers showed an increased level of circulating IL-1ß due to NLRP3-inflammasome induction and an accelerated cardiac fibrosis. The atheroprotective effect of NLRP3 inflammasome inhibitors, as well as their protection against the development of heart failure in *TET2*-deficient mice, supports these findings [26, 39].

### ASXL1

*ASXL1* (additional sex combs-like 1) is the third most mutated gene in CHIP (approximately 5 to 10%), leading to altered histone modification [4••]. *ASXL1* deletion facilitates aberrant gene expression and results in myeloid transformation, but the mechanisms by which *ASXL1* mutations lead to increased inflammation are not entirely clear [27, 40]. Observational studies point to a link between *ASXL1* mutations in blood cells with smoking and among patients with HIV. Dawoud et al. utilized whole-exome sequencing data of the UK Biobank and found a significantly higher risk of having CHIP for those who were current or former smokers [17]. The majority of participants (69%) with an *ASXL1* CHIP were current or former smokers confirming that mutations in *ASXL1* and genes coding for spliceosomes are strongly associated with exposure to DNA-damaging agents such as smoking [17].

### JAK2

*JAK2* (activated janus kinase 2) is a non-receptor tyrosine kinase that transmits intracellular signals downstream of cytokine receptors and accounts for a small percentage of all CHIP, which do not only appear in older age groups. *JAK2* tyrosine phosphorylates and activates *TET2* in response to cytokines, linking extracellular signals with epigenetic changes in hematopoiesis. *JAK2* mutations in CHIP tend to occur at a younger age and carry the strongest risk of premature cardiac disease among CHIP variants [4••, 15]. The presence of *JAK2* CHIP carrier status is associated with higher levels of IL-18, and downstream increases in IL-6 production. Inflammation and atherosclerotic disease have been shown to occur in *JAK2* CHIP carriers even in the presence of reduced LDL cholesterol [4••, 8].

The *JAK2V617F* mutation is commonly linked to myeloproliferative neoplasms, and in these diseases, it is associated with thromboembolic complications, increased blood viscosity, and platelet adhesion, as well as reduced venous blood return [41, 42]. Indeed, the *JAK2*-gain-of-function mutation has been shown to promote the risk of venous and coronary thrombosis and pulmonary embolus, due to its enhanced formation of neutrophil extracellular traps, components of innate immunity [43] Importantly, however, the *JAK2V617F* mutation was also found to be associated with thrombosis in patients without the presence of myeloproliferative neoplasms or other hematologic disorders [43]. Moreover, Wang et al. showed that the *JAK2V617F* mutation expression promotes neutrophil infiltration and early atherosclerotic lesion formation and plaque instability in a mouse model of hypercholesterolemia [44]. Edelmann et al. further evaluated the role of *JAK2V617F* mutation in thrombus formation and found that the mutation upregulates β_1_ and β_2_ integrin expression, which are both essential regulators for attachment of leukocytes to endothelial cells [45]. Collectively, current evidence suggests that *JAK2* mutations in CHIP can promote CVD by altering hematopoiesis-boosting innate immunity responses, and promoting thrombotic diseases [46].

### TP53, PPM1D, SF3B1, SRSF2

Mutations in DNA damage repair genes such as *TP53* or *PPM1D* are less frequent than other CHIP mutations [4••]. In case of a detected DNA damage, the activated tumor suppressor p53 induces the expression of *PPM1D* protein phosphatase (Mn^2+^/Mg^2+^-dependent 1D) which leads to dephosphorylation of p53 and ultimately to apoptosis. Hematopoietic cell lines with *PPM1D* loss-of-function mutations outcompete normal cells by increased resistance to apoptosis and are strongly associated with CHIP after prior exposure to cytotoxic chemotherapies such as cisplatin, etoposide, and doxorubicin [47]. Thus, especially in the case of chemotherapy treatment of solid tumors, hematopoietic mutations in *TP53* and *PPM1D* appear to promote the outgrowth of clones that can lead to subsequent malignancy and risk for leukemic transformation [47, 48].

Mutations of the mRNA spliceosome complex components *SF3B1* and SRSF2 are not well studied yet, but seem to be the most common genetic alterations in patients with myelodysplastic syndrome [49, 50]. Mutations in the splicing factors *SF3B1* and *SRSF2* have been reported to share convergent effects on aberrant splicing of mRNAs that promote nuclear factor κB signaling [50].

### CHIP Without Driver Mutations

CHIP is commonly defined as somatic mutation with variant allele frequency > 2% in peripheral blood of individuals with no evidence of hematologic disease.^47, 48^ Thus, CHIP without known candidate driver mutations is technically excluded from this classification [51, 52]. Nonetheless, clonal hematopoiesis without driver mutations carries increased risk of hematologic cancers and all-cause mortality, although its links to CVD are poorly understood [5, 53]. In fact, in a significant proportion of cases of clonal hematopoiesis, no clear candidate driver mutation is identified [4••, 5]. Mosaic chromosomal alteration represents one presentation of CHIP without driver mutations. It includes larger structural somatic alterations such as deletions, duplications, or copy number neutral loss of heterozygosity [54, 55]. Similar to candidate driver mutations, mosaic chromosomal laterations are common at very old age and have been related to lymphoid malignancies like CLL [56]. Interestingly, cardiovascular risk is not altered in cases of mosaic chromosomal alteration, even when associated with *DNMT3A* or *TET2* loss (with the notable exception of *JAK2*) [8, 54, 55].

### Genotypic Associations with CHIP

While CHIP driver mutations are acquired somatic mutations, certain germline variation may predispose to the development of CHIP during life course. Using whole-genome sequencing data from a large cohort unselected for candidate driver mutation, Bick et al. could identify three germline risk loci associated with a predilection to *TET2* CHIP [4••, 8] One set of loci involved genes that maintain genome integrity (e.g., *TERT* and *CHEK2*) and which have been implicated in stem cell maintenance/self-renewal and the risk of neoplasm in multiple organ systems; other germline loci are associated with increased hematopoietic stem cell self-renewal (e.g., *TET2*) and only associated with hematologic malignancies; finally, a third set of germline loci are associated with the acquisition of CHIP mutations in specific driver genes. Specifically, variations at the *TCL1A* promoter were associated with increased risk of *DNMT3A* CHIP, but not other CHIP subsets [4••, 8].

## CHIP and Age-Related Cardiovascular Risk

In a seminal paper on “Clonal Hematopoiesis and Risk of Atherosclerotic Cardiovascular Disease,” Jaiswal et al. showed that presence of CHIP in peripheral blood cells was associated with accelerated atherosclerosis and coronary heart disease [15, 22]. Subsequent studies have expanded our understanding on CHIP carrier status and CVD (Table 2). Reports on associations of CHIP with multiple other age-related cardiovascular conditions such as heart failure, stroke, or aortic valve stenosis have been published to this point [13, 57, 58] (Fig. 1).

**Table 2:** Key message

Clonal Hematopoiesis of Indeterminate Potential (CHIP) is related to age-related cardiovascular disease.
Age, germline variants, environmental factors and lifestyle exposures have been shown to induce somatic mutations and the development of CHIP.
The most commonly mutated candidate driver genes are *DNMT3A*, *TET2*, *ASXL1* and *JAK2* which are related to inflammatory pathways.

### Coronary Heart Disease

There is now consistent evidence for an epidemiological association between presence of CHIP and myocardial infarction or coronary revascularization procedures [15]. In experimental analysis, it could be further shown that clonal hematopoiesis associated with TET2 deficiency leads to accelerated atherosclerosis mainly driven by interactions between clonal monocytes-macrophages and the endothelium and an increased expression of pro-inflammatory genes [15, 39, 59, 60]. Moreover, RNA sequencing of cells with loss-of-function mutations in *TET2* showed augmented expression of pro-inflammatory mediators implicated in the pathogenesis of atherosclerosis including IL-1β and IL-6 [15].

### Heart Failure

In addition to the relationship between CHIP and coronary heart disease, CHIP has been recently associated with ischemic heart failure and reduced left ventricular ejection fraction [13, 61]. However, even outside the context of ischemic events, CHIP was found to be associated with deteriorating cardiac function. In fact, the presence of CHIP mutations in patients with chronic heart failure has been identified as an independent predictor of mortality [62]. Interestingly, current evidence suggests that heart failure patients carrying more than one CHIP mutation have a worse prognosis; higher cumulative clone size is also an adverse prognostic factor, supporting a dose–response relationship [63, 64].

The underlying mechanisms by which CHIP and its mutations in *ASXL1*, *DNMT3A*, *TET2*, or *JAK2* are related to heart failure development and progression are not well understood. It appears that different CHIP mutations may exert this effect through different signaling pathways and inflammatory profiles. *ASXL1*, *TET2*, and *JAK2* sequence variations have been each associated with an increased risk of heart failure, whereas the association of *DNMT3A* with heart failure shows inconsistent results across studies.

Additional information on the pathways by which the *DNMT3A* or *TET2* mutations alter cardiac function came from Sano et al. who investigated the potential mechanisms in a murine model using a CRISPR/Cas9 system [37]. The researchers used a lentiviral vector to deliver Cas9 and guide RNA, introducing inactivating mutations in *TET2* and *DNMT3A* in bone marrow cells using a model of hypertensive heart failure. Interestingly, only mice with inactivating mutations in *TET2* had expanded mutant hematopoietic cells and increased expression of IL-1B and IL-6, whereas mice with inactivating mutations in *DNMT3A* did not demonstrate expansion of hematopoietic cells. Other data showed that circulating monocytes of patients with heart failure carrying *DNMT3A* mutations demonstrated a pro-inflammatory transcriptome with significantly increased expression of inflammatory genes compared with monocytes derived from patients with heart failure without *DNMT3A* mutations, especially inflammatory interleukin IL-1β, IL6, IL8, the inflammasome NLRP3, and the macrophage inflammatory proteins CCL3, CCL4, and resistin, of which the latter mediates monocyte-endothelial adhesion and may all together contribute to an aggravation of chronic heart failure [65].

### Aortic Valve Stenosis

Mas-Peiro et al. examined the incidence of CHIP in patients with severe degenerative aortic valve stenosis [57]. It appeared that CHIP prevalence was enriched among patients with severe aortic stenosis, as somatic *DNMT3A* or *TET2* driver mutations were detected in a third of patients. CHIP carriers had an increased mortality rate following TAVI procedure, were found to have increased inflammatory activation of T-cells and demonstrated higher circulating levels of non-classical monocytes that secrete pro-inflammatory cytokines, including TNF-α, IL-1β, and IL-8.

### Stroke

CHIP was recently found to be associated with a 14% increased odds of incident stroke when analyzed across eight cohorts [58]. Interestingly and unexpectedly, this relationship was primarily driven by 24% increased odds of hemorrhagic stroke. Unselected subtypes of ischemic stroke were not associated with CHIP. However, in further analyses of ischemic stroke subtypes, CHIP was strongly associated with small vessel stroke, with stronger relationships with mutations in *TET2*. The mechanisms linking CHIP to hemorrhagic stroke are not clear, but again inflammatory signaling pathways linked to aneurysm formation, accelerated arteriosclerosis, blood vessel fragility, and cerebral amyloid angiopathy are potentially involved [58, 66–68].

## CHIP Implications: a New Target for Cardiovascular Disease Prevention

CHIP has been identified as a major, non-lipid/non-traditional mediator of cardiovascular risk. It has been linked to multiple cardiovascular outcomes including coronary heart disease, heart failure, stroke, and aortic valve disease. The underlying pathophysiological mechanisms are most likely related to inflammatory pathways. These exciting findings raise several questions:

*Should cardiovascular risk assessment and management include screening for CHIP in light of recent CHIP findings?* Diagnosing CHIP requires deep sequencing of peripheral blood and it is not yet a routine clinical test. However, as methods for assaying CHIP become more accessible and cost-efficient and as additional treatments targeting CHIP become available, screening for CHIP status may become a routine part of clinical care. Assessing CHIP status offers promise for advancing individualized precision medicine, with the ultimate aim of preventing the accumulation of acquired somatic mutations, atherosclerotic lesion development, and progression. Research on CHIP represents an extension of the study of cardiovascular genetics and atherosclerosis biology beyond inherited germline mutations [6, 69]. Clinical management of individuals with CHIP remains limited, with few treatment strategies beyond traditional risk factor modification. As new data emerge, international recommendations for diagnosis, management algorithms, and treatment options for CHIP can be promulgated.

*How may clinicians be able to leverage CHIP data in the future and what treatment strategies may be indicated ?* Screening for CHIP and its major driver mutations can help clinicians to identify patients at high-cardiovascular risk warranting more intensified lifestyle-related or even pharmacologic interventions. ﻿One potential intervention in patients with CHIP could be to specifically target certain inflammatory pathways. Modulation of IL-1β in the CANTOS trial and IL-1 and Il-8 with colchicine in COLCOT may therefore provide potential tools and stimulate the development of future targeted interventions [40, 70, 71]. Genetically reduced IL-6 signaling in *DNMT3A* and *TET2* CHIP carriers has been shown to substantially reduce CVD risk [72]. Additionally, most recent data from the CANTOS trial raise the possibility that in individuals with established CVD and elevated high-sensitivity C-reactive protein level above 2.0 mg/L, those with *TET2* variants may respond better to Canakinumab, an anti-IL-1β antibody, with respect to CVD event reduction than those without CHIP [73•]. Other suggested therapeutic approaches involve inhibition of *JAK2* or downstream integrins, which may reduce thrombotic CVD complications in CHIP patients carrying *JAK2* mutations, or tight control of glucose levels to mitigate CVD risk in patients with *TET2-*driven CHIP [43, 46, 74, 75].

In conclusion, the recognition of a strong link between somatic mutations, clonal hematopoiesis, and age-related cardiovascular risk provides new insights into the pathophysiology of atherothrombotic cardiovascular conditions and novel approaches to prevention and treatment. This exciting field may have future implications for clinical practice and population health.
